# Towards Rational Computational Engineering of Psychrophilic Enzymes

**DOI:** 10.1038/s41598-019-55697-4

**Published:** 2019-12-16

**Authors:** Jaka Sočan, Geir Villy Isaksen, Bjørn Olav Brandsdal, Johan Åqvist

**Affiliations:** 1 Department of Cell and Molecular Biology, Uppsala University, Biomedical Center, Box 596, SE-751 24 Uppsala, Sweden; 2 Hylleraas Centre for Quantum Molecular Sciences, Department of Chemistry, UiT – The Arctic University of Norway, N9037 Tromsø, Norway

**Keywords:** Enzymes, Theoretical chemistry

## Abstract

Cold-adapted enzymes from psychrophilic species achieve their high catalytic efficiency at low temperature by a different partitioning of the activation free energy into its enthalpic and entropic components, compared to orthologous mesophilic enzymes. Their lower activation enthalpy, partly compensated by an increased entropic penalty, has been suggested to originate from changes in flexibility of the protein surface. Multiple sequence alignments of psychrophilic and mesophilic enzymes also show characteristic motifs located in surface loops of the protein. Here, we use computer simulations to examine the effects of a number of designed surface mutations of psychrophilic and mesophilic elastases on the temperature dependence of the catalyzed peptide cleavage reaction. For each of 14 mutant enzyme variants we report calculations of their thermodynamic activation parameters. The results show that substitution of psychrophilic loop residues into the mesophilic enzyme consistently changes both the activation parameters and loop flexibilities towards the former, and vice versa for opposite substitutions.

## Introduction

Psychrophiles are extremophilic organisms that are able to thrive in the Earth’s coldest ecosystems and they have adapted to withstand the major challenges of low temperature environments. These species have evolved to manifest specific genetic traits enabling them to maintain cellular metabolism under conditions of profoundly lower rates of biochemical reactions and higher water viscosity encountered at low temperature^1^. Besides adaptations involving membrane composition, expression of antifreeze proteins etc., the most remarkable characteristic is the ability of psychrophilic enzymes to mitigate the loss of catalytic activity near the freezing point of water, where their meso- and thermophilic counterparts are usually almost totally inactive^1–3^. This is generally achieved by redistributing the enthalpic and entropic components of the free energy barrier of the catalyzed reaction, in effect moving part of the enthalpic penalty to an entropy penalty^2–7^. Such a redistribution is favorable, since it is the enthalpy difference between the transition state and ground state that gives rise to the exponential decay of reaction rates (${e}^{-\Delta {H}^{\ddagger }/RT}$) as the temperature is lowered. The entropic contribution to the reaction rate (${e}^{\Delta {S}^{\ddagger }/R}$) is instead a multiplicative factor that is independent of temperature, according to standard transition state theory.

One of the earliest studies^8^ comparing highly conserved orthologs of lactate dehydrogenase in fish species with different body temperatures, showed that psychrophilic enzymes have improved kinetic properties at lower temperatures. A putative link to higher conformational flexibility than their mesophilic counterparts was also suggested, based on the observation of somewhat higher *K*_M_ values^8^. A similar coincidence of catalytic activity and enzyme flexibility has been described in various other psychrophilic-mesophilic enzyme pairs^4,5,9–13^. It is interesting to note that the majority of these observations do not report a uniform increase in flexibility throughout the cold-adapted enzyme, instead the largest differences are usually found outside the active site region^9–13^. This indeed makes sense, since active site residues are less likely to diverge among orthologs due to the prohibitive impact on enzyme function of mutations in this region^14^.

The ground-breaking discovery of the lower $\Delta {H}^{\ddagger }$ and more negative $\Delta {S}^{\ddagger }$ values in cold-adapted species by Somero and coworkers^6^ was based on comparisons of the kinetics of lactate dehydrogenase, glyceraldehyde-3-phosphate dehydrogenase and glycogen phosphorylase from mammals and from fish and lobster. Since then, a large number of natural cold-adapted enzymes have been characterized which confirm the activation enthalpy-entropy rule without exceptions, when compared to their mesophilic and thermophilic orthologs^1–13^. It is, however, interesting to note that engineered mesophilic enzymes can be made more efficient at low temperature also by other mechanisms. That is, a recent mutational study of mesophilic *E. coli* adenylate kinase (Adk) showed that the enzyme could be made more efficient at all temperatures by two glycine substitutions in AMP binding domain^15^. Interestingly, these mutations did not affect $\Delta {H}^{\ddagger }$ but reduced the $-T\Delta {S}^{\ddagger }$ penalty by ~0.8 kcal/mol (at 25 °C), corresponding to a 2.5 cal/mol/K more positive activation entropy. As the rate-limiting step in Adk is product release, and not the chemical step, the result was interpreted in terms of a locally (partially) unfolded state of AMP binding domain involved in the transition state for product release^15^, which would reduce the associated entropy penalty. Since the only kinetic effect of the two mutations was on $\Delta {S}^{\ddagger }$ the improved efficiency compared to the wildtype was, however, more pronounced at high than at low temperatures.

There appear to be no simple structural rules for how cold-adaptation of enzymes emerges during evolution. It is clear, however, that cold environments exert a considerably reduced selective pressure on protein thermal stability, which is a predominant survival factor for meso- and thermophilic species^4,16^. As a consequence, psychrophilic proteomes are generally found to be more heat-labile^17^. This is presumably largely due to random genetic drift, since stabilizing interactions between amino acid sidechains will then gradually vanish and increase protein structural flexibility^3,7^. Some accumulated mutations may thus have proven beneficial and been selected for when survival at low temperatures was at stake, even though they might also have reduced thermal stability, provided that the working conditions were already far from the melting temperature of the enzyme. Reported observations^3,5,8^ of moderately increased values of *K*_M_ are also consistent with this idea since raised *K*_M_ values do not confer any catalytic advantage, but are rather detrimental to reaction velocity at small substrate concentrations (*v* ∝ *k*_cat_/*K*_M_), and may thus just be a side-effect of reduced thermal stability. Alternatively, there might in some cases actually be a trade-off between *k*_cat_ and *K*_M_ since a locally more flexible enzyme appears to be a requirement for reducing the activation enthalpy of the catalytic reaction^12^. However, increased *K*_M_ values for cold-adapted enzymes are not a general rule and, e.g., both trypsin and elastase show a lower *K*_M_ for the psychrophilic salmon enzyme compared to mesophilic orthologs^18,19^. From experimental data it is also evident that the major adaptation effect responsible for high activity at low temperature is on *k*_cat_^3,5,8,9^. Interestingly, a review of different psychro- and mesophilic orthologous enzyme pairs examined different possible structural descriptors for how cold-adaptation might be achieved^3^, but no structural features were found to consistently correlate with cold-adaptation. A recent mutational study of a psychrophilic β-galactosidase also showed temperature-dependent changes in *k*_cat_ and *K*_M_ originating from different types of amino acid substitutions^20^.

It has recently become clear that computer simulation methods can be particularly useful for explaining the origins of cold-adaptation of enzymes, since they can provide detailed insight at the microscopic level^7^. Hence, exploration of the enzyme conformational space by molecular dynamics (MD) simulations gives direct information on protein flexibility that is very difficult to obtain by other means. Such data can be used to pin-point regions of significantly different structural mobilities between enzyme orthologs^9–13,21,22^. However, in order to connect such mobility differences to catalytic activities it is necessary to employ computational methods that can couple conformational sampling to the energetics of the enzyme catalyzed reaction. In particular, computational evaluation of thermodynamic activation parameters is critical, since it may establish direct relationships between these parameters and key structural features at different temperatures^9,11,23^. Empirical valence bond (EVB) simulations^24,25^ have so far proven to be the only viable alternative for determination of $\Delta {H}^{\ddagger }$ and $T\Delta {S}^{\ddagger }$, by extensive sampling of free energy profiles leading to the rate-limiting transition state at different temperatures. Remarkably, computational studies applying the EVB method to orthologous variants of citrate synthase^9^, triosephosphate isomerase^10^, trypsin^11^ and elastase^13^, have all elicited the typical redistribution of enthalpic and entropic contributions to the activation free energy in cold-active enzymes, compared to their mesophilic counterparts. Moreover, the origin of the characteristic activation enthalpy-entropy balance in cold-adaptation could in these cases be understood and traced to the altered flexibility of enzyme surface loops^12,26^.

Sequence comparisons of orthologous enzymes have further revealed different conserved characteristic motifs among psychrophilic and mesophilic species, often located at the enzyme surface^5,11,13^. This now raises the interesting possibility to rationally engineer both psychrophilic and mesophilic enzymes to increase their activity at higher and lower temperatures, respectively. To explore whether this could be done solely based on computer simulations, a number of putative hot-spots for mutations related to temperature adaptation were identified for differently adapted elastases. Herein we report extensive EVB/MD free energy calculations on 14 such cases, 7 for each of the two orthologous enzymes salmon (SPE) and porcine pancreatic elastase (PPE). The mutations all substitute the psychrophilic residues with mesophilic ones, and vice versa, and involve both single, double and triple substitutions. We examine the effects on both the thermodynamic activation parameters and on surface loop flexibilities, in order to try to establish a reliable connection between these properties. Interestingly, the results clearly illustrate how changes in a single surface loop can affect the temperature adaptation properties of an enzyme by alteration of the loop mobility.

## Results

### Effects of mutations on thermodynamic activation parameters and rates

**Figure 1:**
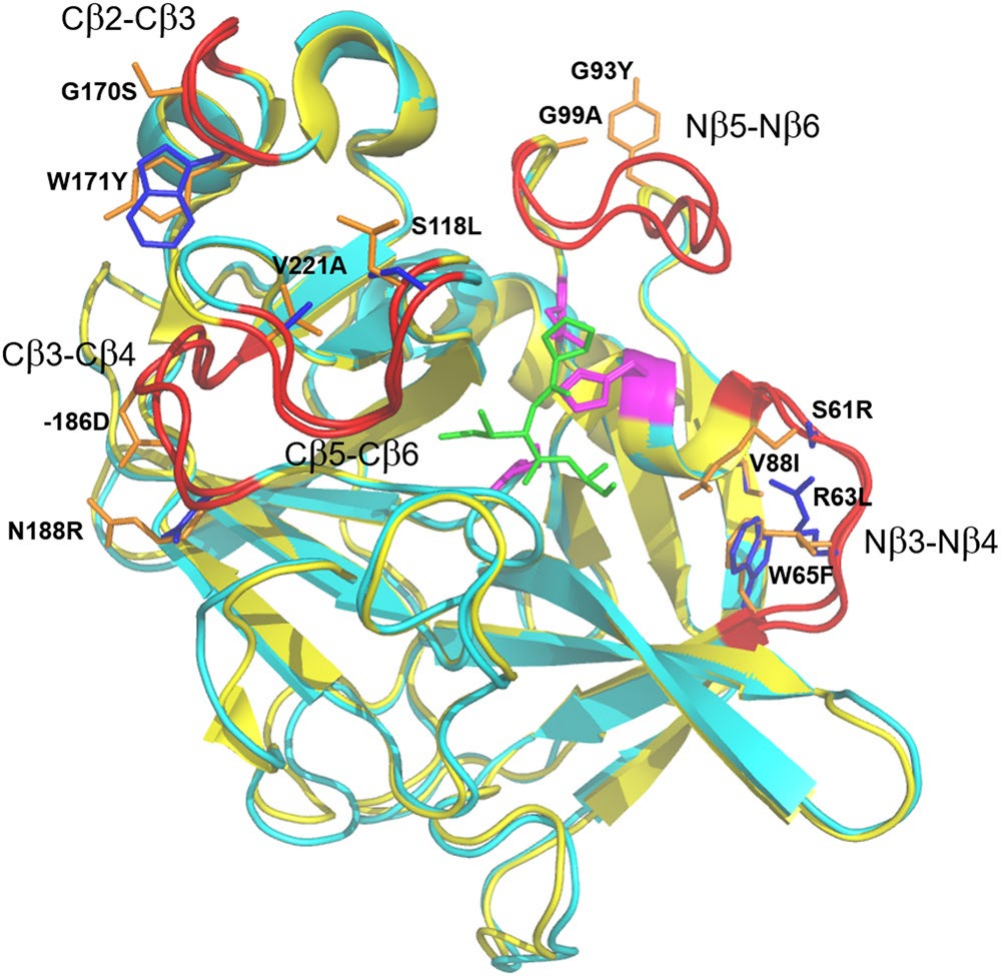
Structures of psychrophilic and mesophilic pancreatic elastases. Crystal structures of salmon (cyan) and porcine (yellow) pancreatic elastases^30–32^, with a docked tripeptide substrate (green) and the catalytic triad coloured in purple. Surface loops that differ in terms of mobility^13^ and where mutations have been selected are coloured in red. Residues mutated in this work are coloured in blue and orange for the salmon and porcine enzymes, respectively, and the mutations are indicated for the salmon enzyme.

**Figure 2:**
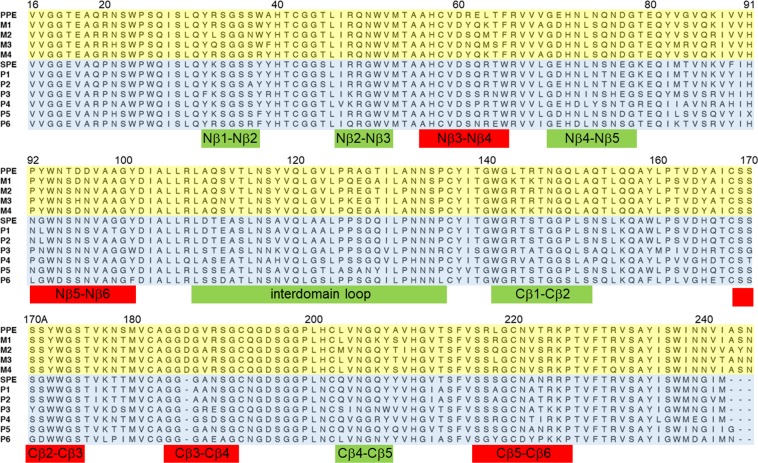
Alignment of mesophilic and psychrophilic elastase sequences. PPE – *Sus scrofa*, M1 – *Pan troglodytes*, M2 – *Erinaceus europaeus*, M3 – *Physeter catodon*, M4 – *Homo sapiens*, SPE – *Salmo salar*, P1 – *Oncorhynchus mykiss*, P2 – *Oncorhynchus kisutch*, P3 – *Notothenia coriiceps*, P4 – *Clupea harengus*, P5 – *Esox lucius*, P6 – *Labrus bergylta*. Mutated loop regions are indicated in red.

Molecular dynamics simulations of the reactant substrate complexes with the salmon and porcine elastases have identified five different loop regions of the two enzymes that differ significantly in terms of mobility^13^. These are the Nβ3-Nβ4, Nβ5-Nβ6, Cβ2-Cβ3, Cβ3-Cβ4 and Cβ5-Cβ6 surface loops (Fig. 1), where the numbering denotes which β-strands are connected and whether the loop is situated in the N- or C-terminal domain of the protein. Sequence comparisons of seven psychrophilic and five mesophilic vertebrate pancreatic elastases also revealed that these loops regions have distinct amino acid signatures in the cold- and warm-active enzymes (Fig. 2). It would hence seem possible that mutations of the conserved positions in these loops could render the psychrophilic salmon enzyme more mesophilic-like and vice versa for mutations at these positions in the porcine elastase. In fact, our earlier MD/EVB calculations^13^ tested this hypothesis in a preliminary way for the S218L/A221V double mutant of SPE and for the reverse L218S/V221A variant of PPE. For the Pro-Ile-Ala tripeptide substrate this yielded computed values of $\Delta {H}^{\ddagger }=11.3$ and $T\varDelta {S}^{\ddagger }=-\,6.7$ kcal/mol for the SPE double mutant and rather similar values, $\varDelta {H}^{\ddagger }=11.4$ and $T\varDelta {S}^{\ddagger }=-\,8.0$ kcal/mol, for the PPE mutant. This is quite remarkable since the two wildtype enzymes had predicted activation parameters of $\varDelta {H}^{\ddagger }=4.6$ and $T\varDelta {S}^{\ddagger }=-\,13.4$ kcal/mol (SPE) and $\varDelta {H}^{\ddagger }=17.2$ and $T\varDelta {S}^{\ddagger }=-\,2.0$ kcal/mol (PPE), respectively, with differences typical for adaptation to different temperatures^13^. Hence, the two double mutants indeed gave each enzyme intermediate characteristics between the psychrophilic and mesophilic extremes. It may also be noted that the large differences (>10 kcal/mol) in $\varDelta {H}^{\ddagger }$ and $T\varDelta {S}^{\ddagger }$ between the two wildtype enzymes are similar to those obtained experimentally for the related serine protease trypsin^27^. In that case values of $\varDelta {H}^{\ddagger }=9.7$, $T\varDelta {S}^{\ddagger }=-\,7.1$ kcal/mol and $\varDelta {H}^{\ddagger }=19.7$, $T\varDelta {S}^{\ddagger }=+\,2.7$ kcal/mol were reported for the Antarctic cod and bovine enzymes, respectively^27^. Our earlier MD/EVB simulations^11^ of the psychrophilic salmon and bovine enzymes gave $\varDelta {H}^{\ddagger }=9.9$, $T\varDelta {S}^{\ddagger }=-\,8.3$ kcal/mol and $\varDelta {H}^{\ddagger }=20.4$, $T\varDelta {S}^{\ddagger }=+\,1.4$ kcal/mol, which shows that the calculations are indeed reliable enough to capture these effects. The accuracy of computationally derived Arrhenius plots from MD/EVB simulations is further discussed in ref. ^26^ for various test cases, including both solution and enzyme reactions as well as GTP hydrolysis by EF-Tu on the ribosome.

To further test the role of conserved sequence motifs in the psychrophilic and mesophilic enzymes, and to explore whether it could actually be possible to engineer temperature adaptation properties in rational way, we selected twelve additional mutants in the above mentioned loops, six for each enzyme. For these mutants we carried out extensive MD/EVB simulations of their catalytic reactions at five different temperatures ranging from 12 °C to 32 °C. The SPE mutants were S61R, R63L/W65F/V88I, G93Y/G99A, G170S/W171Y, −186D (Asp insertion), and −186D/N188R. Conversely, R61S, L63R/F65W/I88V, Y93G/A99G, S170G/Y171W, D186− (Asp deletion) and D186−/R188N were chosen for PPE. They thus correspond to single, double and triple mutations of the two enzymes and the distances from the substrate carbonyl carbon to the Cβ atoms of the mutated residues range between 14 and 24 Å. In other words, the mutations are rather distant to the active site (Fig. 1), which is in line with the notion that active site residues cannot be touched by evolution if they are already optimized for the catalytic reaction. The mutated positions were chosen based on the presence of different conserved amino acids in psychrophilic and mesophilic sequences (Fig. 2). An exception, however, are the S61R (SPE) and R61S (PPE) mutations where Ser61 appears strictly conserved in psychrophilic variants, while the position is more variable in mesophilic sequences and Arg is specific for the porcine enzyme. The S61R and R61S mutations were, nevertheless, chosen here because the Arg61 sidechain in PPE is the one that comes closest to the reactive center (~8 Å from the substrate carbonyl carbon), among mutations between the two enzymes. For each mutant we carried out at least 50 independent MD/EVB calculations at the five different temperatures to obtain free energy profiles that allow us to extract the thermodynamic activation parameters via standard Arrhenius plots^7^ (Supplementary Fig. 1).

**Figure 3:**
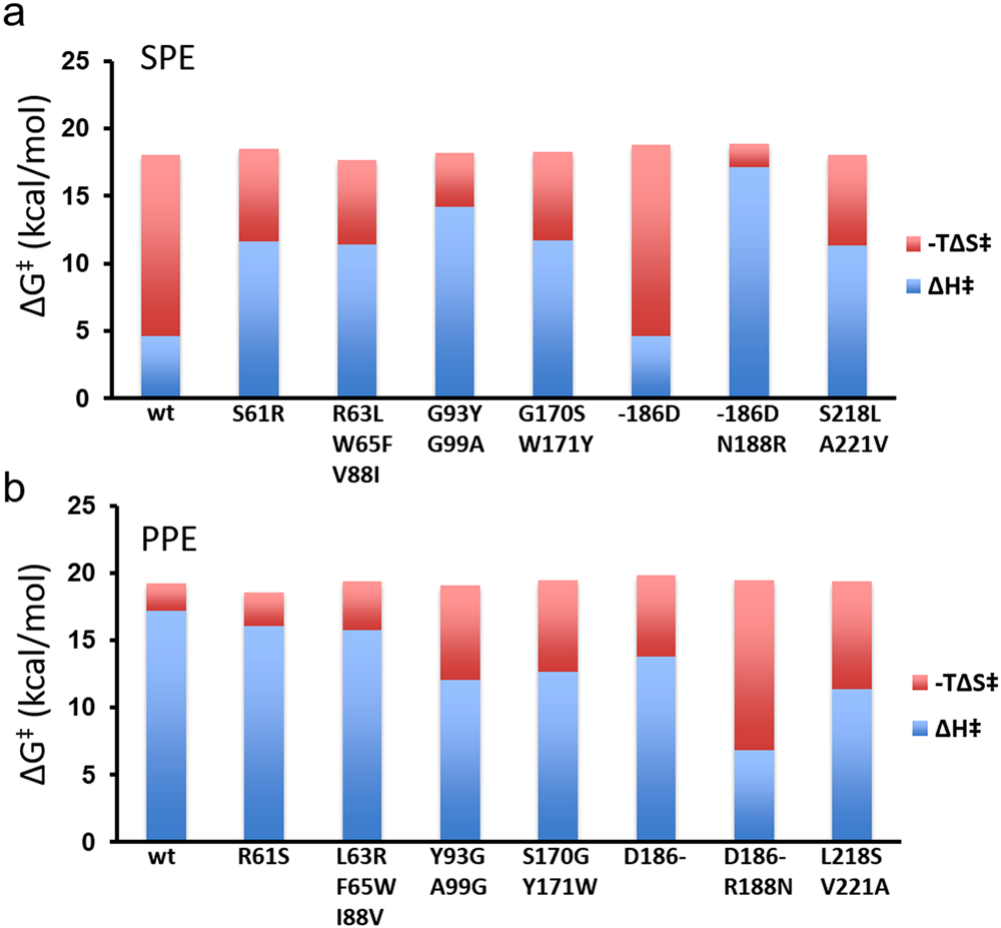
Calculated thermodynamic activation parameters. Values of $\varDelta {G}^{\ddagger }$, $\varDelta {H}^{\ddagger }$ and $-T\Delta {S}^{\ddagger }$ at 22 °C are shown for the rate-limiting step of peptide bond cleavage by wildtype and mutant variants of the salmon (**a**) and porcine (**b**) elastases. The activation parameters were obtained from Arrhenius plots based on MD/EVB calculations of free energy profiles at five different temperatures in the range 12–32 °C.

The results from these computer simulations are shown in Table 1 and Fig. 3 in terms of activation free energies and their enthalpic and entropic components at 22 °C (examples of detailed free energy profiles are shown in Supplementary Fig. 2). Remarkably, all the mutants show pronounced shifts in the activation enthalpy-entropy balance in the expected direction, except for the single D186 insertion in the salmon enzyme. However, although the thermodynamic components are predicted to change by up to ~10 kcal/mol, there is evidently significant enthalpy-entropy compensation at play and the effects on the activation barrier at 22 °C ($\varDelta {G}^{\ddagger }$) are generally just a few tenths of a kcal/mol. This is, of course, in agreement with the general notion that distant mutations usually have small effects on catalytic rates, but what is surprising is that the mutations can have such large impact on the activation enthalpy-entropy balance. It can further be noted that the difference in $\Delta {G}^{\ddagger }$ between the two wildtype enzymes, both from our calculations (Table 1) and experiments with the succinyl-Ala-Ala-Pro-Ile-*p*-nitroanilide substrate^19^, is larger than the effect of any of the mutations of the activation free energy. This shows that amino acid substitutions in a single loop do not suffice for completely changing the temperature adaptation characteristics of one enzyme into the other, although they may provide incremental steps.

**Table 1:** Calculated thermodynamic activation parameters (kcal/mol) for peptide bond hydrolysis by salmon (SPE) and porcine (PPE) pancreatic elastase at 22 °C.

Mutation	SPE			PPE		
	ΔG^‡^	ΔH^‡^	TΔS^‡^	ΔG^‡^	ΔH^‡^	TΔS^‡^
WT	18.0	4.6	−13.4	19.2	17.2	−2.0
S61R	18.5	11.6	−6.9	18.5	16.1	−2.5
R63L/W65F/V88I	17.7	11.4	−6.3	19.4	15.7	−3.6
G93Y/G99A	18.2	14.2	−4.0	19.1	12.0	−7.1
G170S/W171Y	18.3	11.7	−6.5	19.5	12.6	−6.9
−186D	18.8	4.6	−14.2	19.8	13.8	−6.0
−186D/N188R	18.8	17.2	−1.7	19.4	6.8	−12.6
S218L/A221V	18.0	11.3	−6.7	19.4	11.4	−8.0

Since SPE is found to be the faster of the two elastases, both from our calculations and experiments^19^, it can be considered more highly optimized with respect to its catalytic rate. However, none of the PPE mutants are able to reach the same value of *k*_cat_ at low temperature as wildtype SPE, although the R61S, G93Y/G99A and D186−/R188N variants are predicted to increase their activity at 4 °C by a factor of 2–4 (Table 2). Here, the R61S stands out as the only PPE mutation that is more efficient than the wildtype at all temperatures, which is due to its 1.4 kcal/mol lower activation enthalpy. This, in turn, can be traced to destabilizing effect of Arg61 on the protonated His57 of the catalytic triad in the tetrahedral intermediate (TI) of the reaction. Hence, removal of the Arg61 positive charge is predicted to lower the energy of the TI and transition state, as it is closer to His57 than to the Ser195 nucleophile or oxyanion of the TI. It may also be noted that six out of the seven PPE mutants are predicted to gain activity at 4 °C, while four out of seven SPE mutants show increased activity at 39 °C (the average porcine body temperature). What is also interesting is that all the PPE mutants except R61S are predicted from the activation parameters to lose activity at 39 °C, while the SPE mutants are consistently predicted to lose activity at 4 °C (Table 2). Taken together, the results clearly support the idea that these loops are involved in temperature adaptation. It may also be noted that the most versatile enzyme variant is predicted to be the triple R63L/W65F/V88I mutant of SPE, which still shows 71% of the wildtype activity at 4 °C, while the rate is predicted to increase by a factor of 1.5 and 2.9 at 22 °C and 39 °C, respectively (Table 2).

**Table 2:** Calculated catalytic rate constants (×100 s^−1^) for the two enzymes at different temperatures, using transition state theory and assuming constant activation enthalpies and entropies.

Mutation	SPE			PPE		
	*k* (4 °C)	*k* (22 °C)	*k* (39 °C)	*k* (4 °C)	*k* (22 °C)	*k* (39 °C)
WT	15.9	28.9	47.7	0.4	2.8	15.1
S61R	2.9	11.4	36.0	1.5	10.0	48.3
R63L/W65F/V881	11.3	43.7	136.2	0.4	2.5	11.5
G93Y/G99A	3.7	19.3	77.7	1.0	4.0	13.2
G170S/W171Y	4.0	16.2	51.8	0.5	2.0	7.0
−186D	3.6	6.5	10.7	0.2	1.1	4.4
−186D/N188R	0.8	6.0	32.0	0.9	2.1	4.2
S218L/A221V	6.6	25.3	78.0	0.6	2.3	7.2

### The selected mutations consistently affect backbone mobility

**Figure 4:**
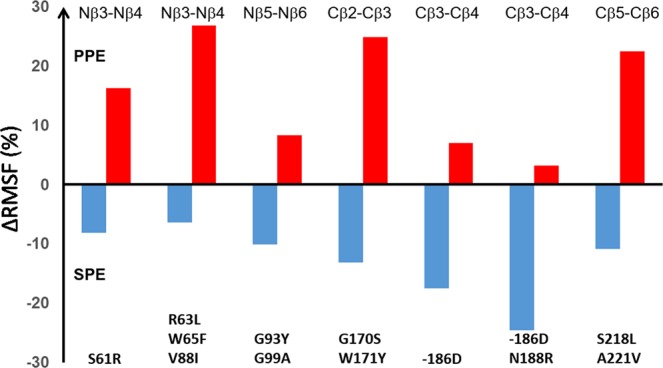
Calculated changes in backbone mobility. Comparison of average backbone positional root-mean-square fluctuations (RMSF) of mutated surface loops relative to the respective wildtype enzymes (SPE – blue, PPE – red). For each mutant in SPE and PPE the change in RMSF relative to the wildtype is given as the percentage of increase or decrease. The calculated values are obtained from four independent 10 ns MD simulations of the reactant state of each system.

The mutations explored in this work were selected based on two criteria, namely (1) the existence of differently conserved motifs in psychrophilic and mesophilic sequence alignments and (2) significantly different calculated backbone mobilities of these loops in the salmon and porcine enzymes^13^ (Supplementary Fig. 3). In general, the average backbone RMSF obtained from MD simulations for the surface loops accommodating the mutations was about 30–40% higher for SPE than PPE^13^. We now examined the effect of the loop mutations on the mobilities of the two proteins and the results are summarized in terms of the average RMSF shifts (in %) for the loops relative to wildtype enzymes in Fig. 4. The most striking result is that all the mutants of SPE show a reduced loop mobility while, conversely, all the mutants of PPE show increased average loop mobilities, precisely as intended. The overall effects range from about 5% to 25% (increase for PPE and decrease for SPE), which indicates that the mutations have captured some, but not all, of the differences between the two wildtype enzymes. However, the fact that all mutations behave as expected in terms of loop mobility is rather remarkable and shows that psychrophilic-mesophilic 3D structure-sequence alignments are indeed interpretable in terms of flexibility, if guided by MD simulations. Moreover, the fact that all mutations behave as predicted both in terms of loop mobilities and in terms of the catalytic activation parameters provides strong direct evidence for a connection between these quantities and their role in cold-adaptation. As noted above, the only exception is the single D186 insertion mutant of SPE, where the activation parameters essentially remain unchanged, and this turns out to be rather instructive from a design point of view.

**Figure 5:**
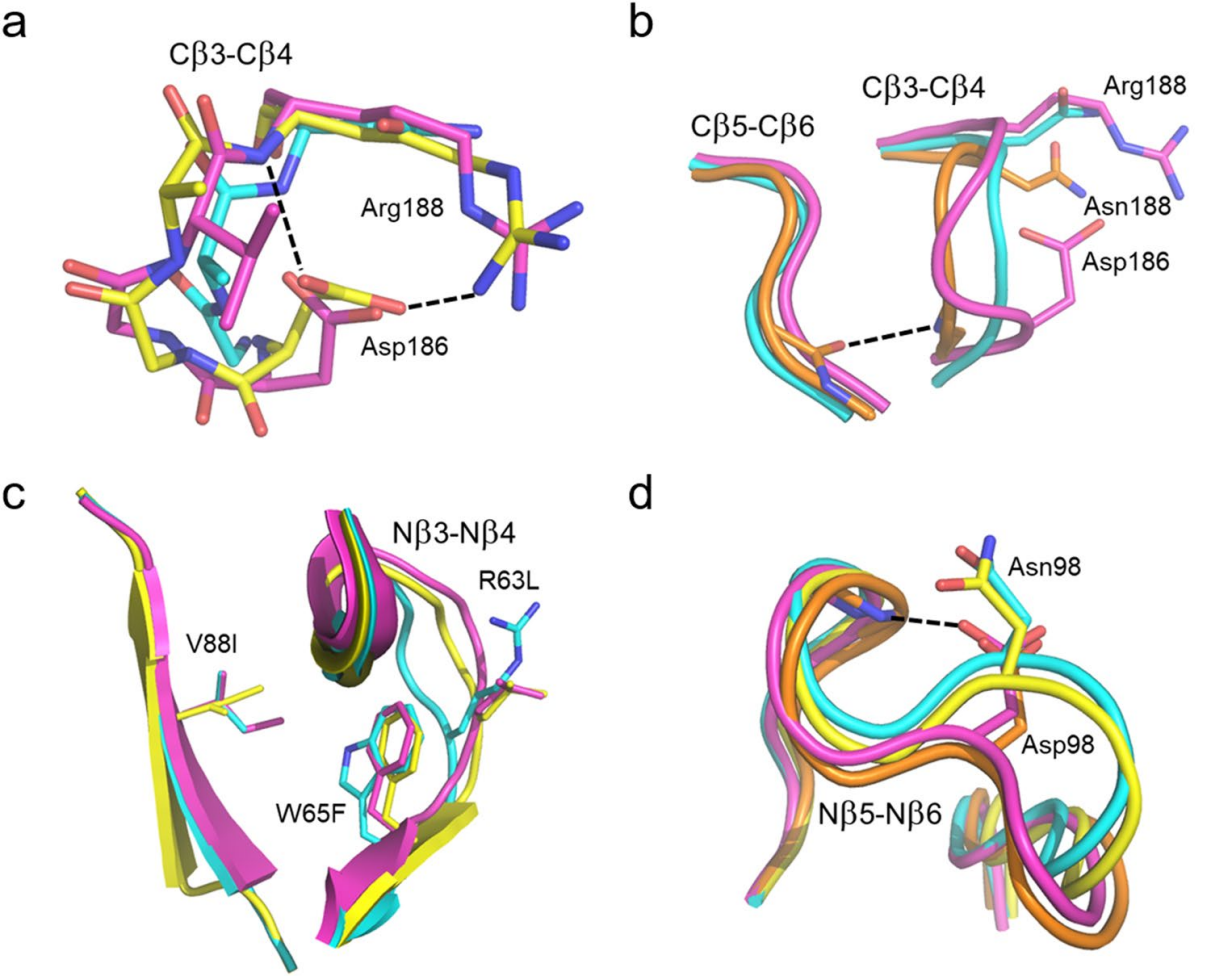
Predicted mutant structures. (**a**) Average MD structure of the −186D/N188R mutant of SPE and (**b**) the reverse D186−/R188N variant of PPE, involving the Cβ3-Cβ4 loop. The salmon and porcine wildtype enzymes are shown with cyan and purple carbons, respectively, while the −186D/N188R mutant is shown in yellow (**a**) and the D186−/R188N mutant in orange (**b**). Key hydrogen bonds are indicated with dashed lines. (**c**,**d**) Average structures of mutants involving the Nβ3-Nβ4 (**c**) and Nβ5-Nβ6 (**d**) loops. The wildtype crystal structures are shown with cyan and purple carbons for SPE and PPE, respectively. The R63L/W65F/V88I (**c**) and G93Y/G99A (**d**) mutants of SPE are show in yellow, while the Y93G/A99A mutant of PPE (**d**) is shown in orange. The backbone H-bond with Asp98 in the two PPE structures is indicated with a dashed line (**d**).

In wildtype PPE the Asp186 residue forms both an ion-pair with Arg188 and a hydrogen bond with the backbone nitrogen of the same residue (Fig. 5a). As Arg188 is an asparagine in SPE, the single Asp186 insertion does not suffice for establishing the same interactions within the loop as in PPE. Hence, although the H-bond with the backbone is indeed formed in the mutant and the RMSF consequently decreases by about 17%, this is apparently not enough for altering the activation enthalpy-entropy balance. In the double SPE mutant −186D/N188R, on the other hand, the ion-pair is also formed (Fig. 5a) which causes both an additional decrease in loop mobility and a concomitant large shift in $\varDelta {H}^{\ddagger }$ and $T\varDelta {S}^{\ddagger }$. It is thus noteworthy that this SPE mutant is clearly the one that mostly resembles the native mesophilic elastase in terms of its activation parameters (Table 1) and it is also the one that has lost most of its catalytic activity at 4 °C (Table 2). Likewise, the reverse mutant D186−/R188N is the PPE variant that most resembles wildtype SPE in terms of its enthalpy-entropy balance. It has also more than doubled its predicted rate at 4 °C and has the lowest rate of all mutants at 39 °C. Nevertheless, the D186−/R188N mutant of PPE shows only a small increase of its loop RMSF, which appears due to formation of an H-bond between the backbones of the Cβ3-Cβ4 and Cβ5-Cβ6 loops (Gly181-Val218), that is not seen in either of native enzymes or the −186D/N188R SPE variant (Fig. 5b). Hence, although the double PPE mutation renders the loop conformation similar to that seen in SPE, the mobility remains lower due to strengthened interactions between the two aforementioned loops.

Although the single −186D insertion in SPE could in retrospect be regarded as a negative control, as it is not predicted to alter the activation enthalpy-entropy balance as discussed above, we also decided to check two additional SPE mutants that were intentionally chosen as negative controls. These are the A121G and S126A mutations which are both located in the interdomain linker on the opposite side of the enzyme from the active site. Here, the former position has identical backbone mobility in the two wildtype enzymes while the latter position has a higher mobility in PPE than SPE, rather than the other way around (RMSF = 1.24 Å compared to 0.74 Å). Moreover, neither of the amino acids at these positions are strictly conserved in the psychrophilic sequences and Ala126 in PPE is not either conserved among mesophiles (Fig. 1b), indicating that these positions are not critical for temperature adaptation. Indeed, the calculated activation parameters for the A121G and S126A mutants of SPE are $\varDelta {H}^{\ddagger }=6.0$, $T\varDelta {S}^{\ddagger }=-12.0$ kcal/mol and ${H}^{\ddagger }=5.7$, $T\varDelta {S}^{\ddagger }=-12.4$ kcal/mol, respectively, which is close to the values obtained for wildtype SPE (Table 1). Also, as expected, neither of the mutations has any significant effect on $\varDelta {G}^{\ddagger }$ at 22 °C.

In general, the structures predicted from the MD simulations of the mutated enzymes offer no major surprises. A relevant example is the R63L/W65F/V88I triple mutant of SPE which, as noted above, is the only SPE variant with predicted activity higher than the wildtype at both 22 °C and 39 °C (Table 2). The MD simulations show that the triple mutation indeed brings the corresponding Nβ3-Nβ4 loop away from its native structure in SPE and very close to the conformation seen in the porcine enzyme. Moreover, the positions and conformations of the three mutated sidechains coincide very well with their observed structures in PPE (Fig. 5c). In contrast, however, the G93Y/G99A double mutant of SPE as well as its Y93G/A99G counterpart in PPE do not cause the Nβ5-Nβ6 loop to adopt the expected target conformations (Fig. 5d). That is, the SPE mutant remains stuck in the wildtype loop conformation and vice versa for the PPE double mutant, which may explain the relatively modest changes in loop mobility for both mutants (Fig. 4). The reason for this behavior turns out to be the existence of a stabilizing intra-loop H-bond in PPE between Asp98 and the backbone nitrogen of Ala99B which is maintained in the simulations of the Y93G/A99G variant of PPE. Apparently, this interaction locks the loop into its native conformation despite the two mutations to glycine. In SPE Asp98 is instead an asparagine which relieves the compact loop conformation in SPE and does not have any H-bonding interaction with the backbone, and hence the G93Y/G99A mutant of SPE does not either reach the PPE conformation because of this (Fig. 5d). The residue Asp/Asn98 was not mutated in our calculations since it is not characteristic of the mesophilic sequences, but was only seen in the porcine sequence, while Asn is otherwise the more common amino acid at this position (Fig. 2). Nevertheless, our calculations predict (Table 2) that the Y93G/A99G mutant of PPE is one that has achieved higher catalytic rates at both 22 °C and 4 °C, without any significant loss of activity in the high temperature regime.

## Discussion

Here we sought to explore the detailed connections between enzyme surface flexibility and cold-adaptation of catalytic rates. We also wanted to test the hypothesis that mutations which would alter the temperature dependence of catalysis could be predicted in a rational way by multiple sequence alignments together with MD simulations. As the key signature of the altered temperature dependence between psychrophilic and mesophilic enzymes is the shift in the activation enthalpy-entropy balance, we calculated free energy profiles for wildtype and mutant elastase at different temperatures and could thereby obtain thermodynamic activation parameters from the corresponding Arrhenius plots. The mutants of psychrophilic salmon elastase were chosen to render the enzyme more mesophilic-like and vice versa for the mesophilic porcine enzyme. Remarkably, out of the 14 mutant variants chosen of the two enzymes, 13 of them showed the expected shift in activation enthalpy and entropy. Moreover, all of the mutant variants showed the expected change in surface loop flexibility (where the mutations were located), with a mobility decrease for the psychrophilic enzyme and an increase for the mesophilic one. Taken together, these results provide unambiguous evidence for role of surface loop flexibility in modulating the temperature adaptation properties.

From the engineering perspective it is noteworthy that all SPE mutants were predicted to lose activity at 4 °C and, but four out of seven were also predicted to gain activity at high temperature. Conversely, for the mesophilic enzyme all mutants except R61S were predicted to lose activity at 39 °C and five out of seven were predicted to improve their efficiency at 4 °C. While this could be considered as an instance of a rule saying that - it is easier to destroy enzyme activity than to improve it - the results are, nevertheless, clearly promising. That is, among the different mutants we have at least one for each enzyme that is predicted to have a significantly higher value of *k*_cat_ away from its working temperature. Hence, the R63L/W65F/V88I mutant of SPE is predicted to be 1.5 and 3 times faster than the wildtype at 22 °C and 39 °C, respectively, while the rate at 4 °C remains essentially unchanged. In PPE the R61S mutation renders the enzyme faster at all temperatures, with the largest rate enhancement (a factor of 4) at 4 °C. Similarly, the Y93G/A99G mutant of PPE is predicted to be 1.4–2.5 faster than the wildtype at the two lower temperatures, without loss of activity at 39 °C. In the latter case, it is also likely that the additional D98N mutation would have further improved its low temperature performance, as discussed above.

A main question to be answered now is, however, why do essentially all the SPE variants have a lower activation free energy (Table 2) than the PPE variants, the only exception being R61S? That is, the 1.2 kcal/mol intrinsic difference in ${\boldsymbol{\Delta }}{{\boldsymbol{G}}}^{\ddagger }$ between the two wildtype catalyzed reactions is basically reflected also by the different mutants and, in particular, none of the PPE variants are able to bring the barrier down to the level seen for the wildtype salmon enzyme. This may have several explanations, where one is that combinations of mutations in several surface loops are required and that these will show cooperative (non-additive) effects on the activation free energy. Another possibility is, of course, that we have missed conserved mutations in some key regions that did not show any mobility differences in the MD simulations. Here, possible candidates would be Trp38 and Asn50 in PPE (Tyr38 and Gly50 in SPE) situated in the Nβ1-Nβ2 and Nβ2-Nβ3 loops, respectively, which did not show any strong RMSF signals in the MD simulations. However, judging from the crystal structures theses mutations could potentially affect hydrophobic packing (W38Y) and hydrogen bonding (N50G) of the two loops.

A third possibility is that conserved mutations among psychrophilic and mesophilic sequences, which are presumably related to temperature adaptation, are not the ones directly responsible for the difference in activation free energy between SPE and PPE at room temperature. In other words, there could be other specific substitutions between the salmon and porcine enzymes, that are not conserved in multiple sequence alignments among psychrophiles and mesophiles, respectively, but that are important for their absolute catalytic rates. The case of Ser61 in SPE points to such a possibility, since the R61S substitution in PPE is predicted to lower the activation free energy at all temperatures. However, while the Ser residue is conserved in psychrophilic sequences, the Arg is not conserved among mesophiles (Fig. 2). A reason for this might be that, when examining the predicted rates at the working temperature of the enzyme, one finds that the porcine elastase has approximately the same rate at 39 °C as the salmon enzyme has at 4 °C. Hence, it may well be the case that there has simply been no evolutionary pressure on further improving the speed of the porcine enzyme. Whatever the answer may be, the present study clearly suggests that a systematic evaluation of mutants, by computational characterization of their catalytic reaction rates at different temperatures, will be very useful for engineering thermal adaptation properties. In this context, molecular dynamics based free energy calculations may also be used to directly assess the effects of mutations on substrate binding affinity and protein stability.

## Methods

### Molecular dynamics simulations

All simulations were performed with the molecular dynamics program Q^28^, using the OPLS-AA force field parameters^29^. The mesophilic porcine elastase (PPE) structure was assembled based on structural information from two Protein Data Bank (PDB) entries, 3HGP^30^ and 1QNJ^31^ as described earlier^13^. The psychrophilic salmon elastase (SPE) structure was based on the PDB entry 1ELT^32^ and was modified to include the correct wild-type amino acid sequence, as discussed in ref. ^13^. Enzyme-substrate complexes were created by adding the tripeptide substrate Pro-Ile-Ala to the active site of each wild-type structure, using the tripeptide docking pose from earlier work^11,13^. Spherical boundary conditions were employed for the MD simulations, where the entire enzyme-substrate complex was solvated by water using a 35 Å radius sphere. Further information on preparation of the wild-type enzyme systems can be found in ref. ^13^.

### Preparation of mutant enzymes

Several mutant systems were prepared to test the impact of each particular surface loop sequence on both its mobility and on the overall thermodynamic activation parameters of the catalyzed reaction. The initial structure for each mutant was based on its corresponding pre-solvated wild-type structure, but with crucial residues in certain surface loops substituted for those found in the wild-type structure of the enzyme with opposite thermal adaptation properties. The mutated residues were inserted into the target wild-type structure so as to obtain a conformation resembling that in the template structure, which was guided by superpositioning of the two native structures of the porcine and salmon enzymes. In addition to the two mutant systems described earlier^13^, twelve new mutants were constructed here (Fig. 1).

### Determination of free energy profiles

The catalytic properties of the mutant enzyme were determined by EVB/MD simulations^24,25^, where a two-state EVB model was used to represent the rate-limiting acylation step of the peptide hydrolysis reaction^13^. The free energy perturbation (FEP) method was used to obtain reaction free energy profiles, using 51 FEP sampling windows for each EVB/MD calculation, which describes the reaction path from the reactant state to the tetrahedral intermediate^13^. The definition of the reacting region and the EVB parameters (gas-phase shift and Hamiltonian off-diagonal coupling element) for the Pro-Ile-Ala substrate were taken from earlier work ($\Delta \alpha =195.0,{H}_{12}=113.0$)^13^. Prior to all MD simulations, the mutant systems were first subjected to a stepwise equilibration procedure, where the system was successively heated from 1 to 295 K in the tetrahedral intermediate state^13^. Reaction free energy profiles for each enzyme system were produced over a range of five different temperatures, 285, 290, 295, 300 and 305 K, where 295 K (22 °C) is the temperature where experimental measurements of kinetic parameters for the succinyl-Ala-Ala-Pro-Ile-*p*-nitroanilide substrate have been reported^19^. At every temperature, at least 50 separate EVB runs were executed per system, each initiated with different randomly chosen atomic velocities according to the Maxwell-Boltzmann distribution at the given temperature. To ensure satisfactory convergence of the thermodynamic activation parameters, the number of replicate simulations per system and temperature were increased by 10 until the Arrhenius plots of $\varDelta {G}^{\ddagger }/T$ versus $\,1/T\,$reached a regression coefficient (*R*^2^) above 0.8. The total simulation time for all EVB/MD simulations was about 2 μs.

### Backbone mobility evaluation

For all 14 mutant systems (Fig. 1), backbone atomic positional fluctuations were analyzed separately from the EVB calculations by executing four independent 12 ns reactant state MD simulations at 295 K. These MD simulations started from the equilibrated EVB simulations, using different initial conditions. The systems were then first allowed to equilibrate in the reactant state for 2 ns, and the mobility analysis was then applied to the last 10 ns of each simulation. The total simulation time here amounted to 560 ns. Average structures based on trajectories of the production phase of each simulation, were calculated using the iterpose functionality of the ProDy MD analysis package and using a framerate of 0.1 ps^33^.

## Supplementary information

Supplementary Information

## Data Availability

The data that support the findings of this study are available from the corresponding author upon reasonable request.
