# Supplementary material for: Towards Rational Computational Engineering of Psychrophilic Enzymes
Source: Sci Rep. 2019 Dec 16;9:19147. doi: 10.1038/s41598-019-55697-4 (PMC6915740; doi:10.1038/s41598-019-55697-4)

Supplementary Information

Towards Rational Computational Engineering of Psychrophilic Enzymes

Jaka Sočan, Geir Villy Isaksen, Bjørn Olav Brandsdal and Johan Åqvist

Supplementary Figure 1. Calculated Arrhenius plots for salmon and porcine elastase mutants. Panels on the left correspond to mutants of the salmon enzyme and panels on the right to mutants of the porcine enzyme.

Supplementary Figure 2. Reaction free energy profiles for selected mutations. Average free energy profiles calculated at 22°C for SPE S61R (black), PPE R61S (red), SPE −186D/N188R (blue) and PPE D186−/R188N (orange). The EVB reaction coordinate is denoted by $\Delta\varepsilon$, the energy gap between the two diabatic potential energy surfaces[24,25]. The s.e.m. for the free energy barriers is ~0.1 kcal/mol.

Supplementary Figure 3. Backbone mobility of salmon and porcine elastases from MD simulations. Positional root mean square fluctuations averaged per residue for the enzyme backbone from 100 ns MD simulations. Results for SPE and PPE are shown in blue and red, respectively, and the trypsin numbering is used.

Supplementary Fig. 1

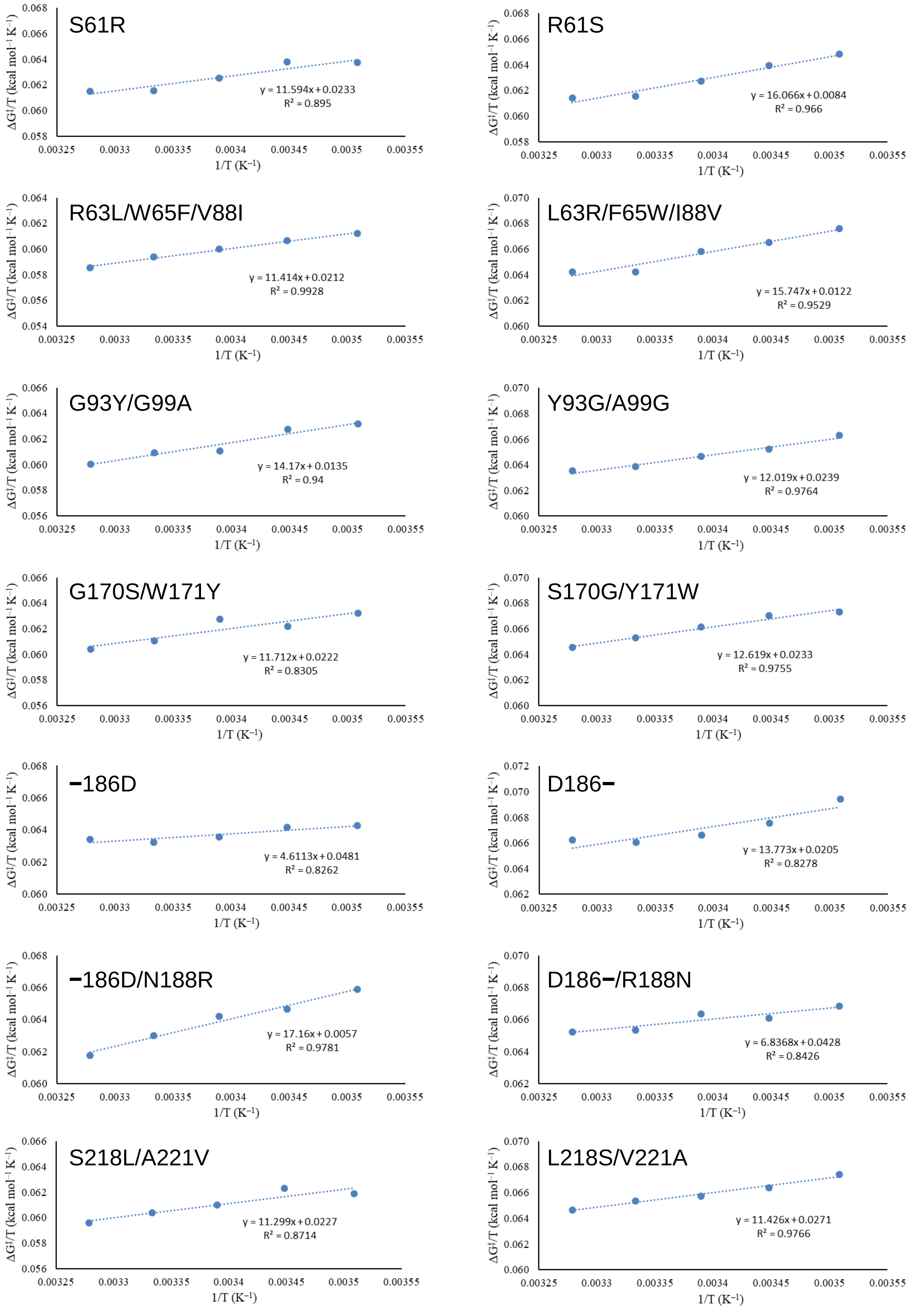

Supplementary Fig. 2

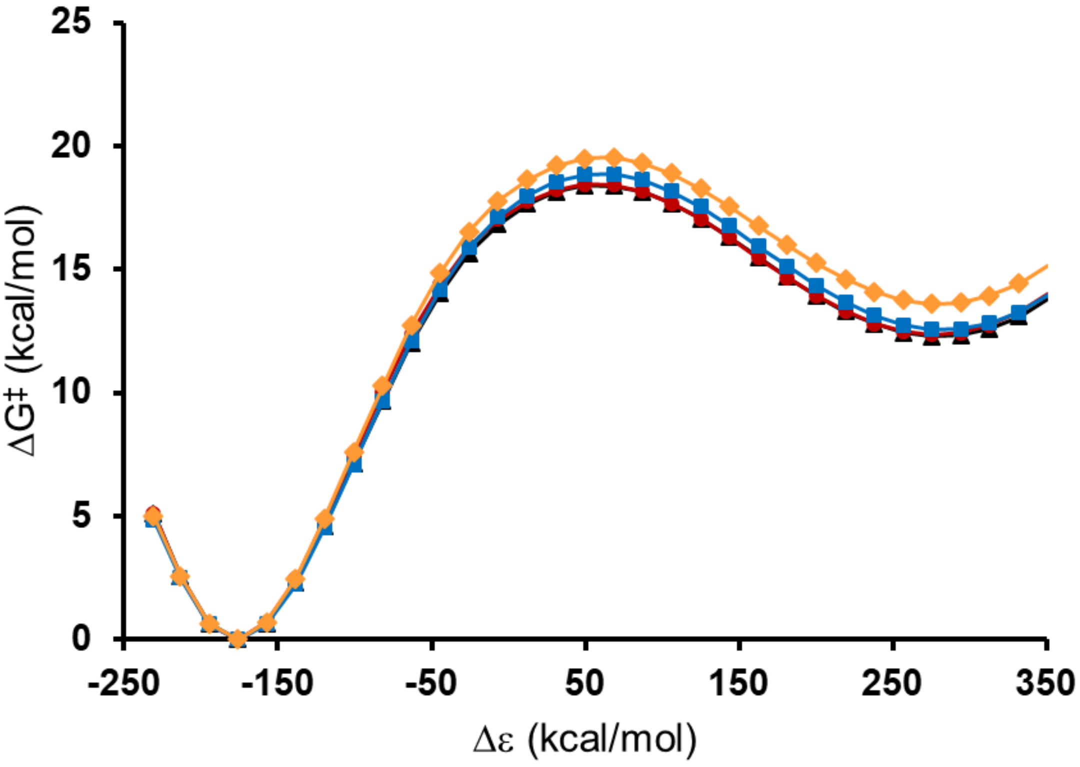

Supplementary Fig. 3

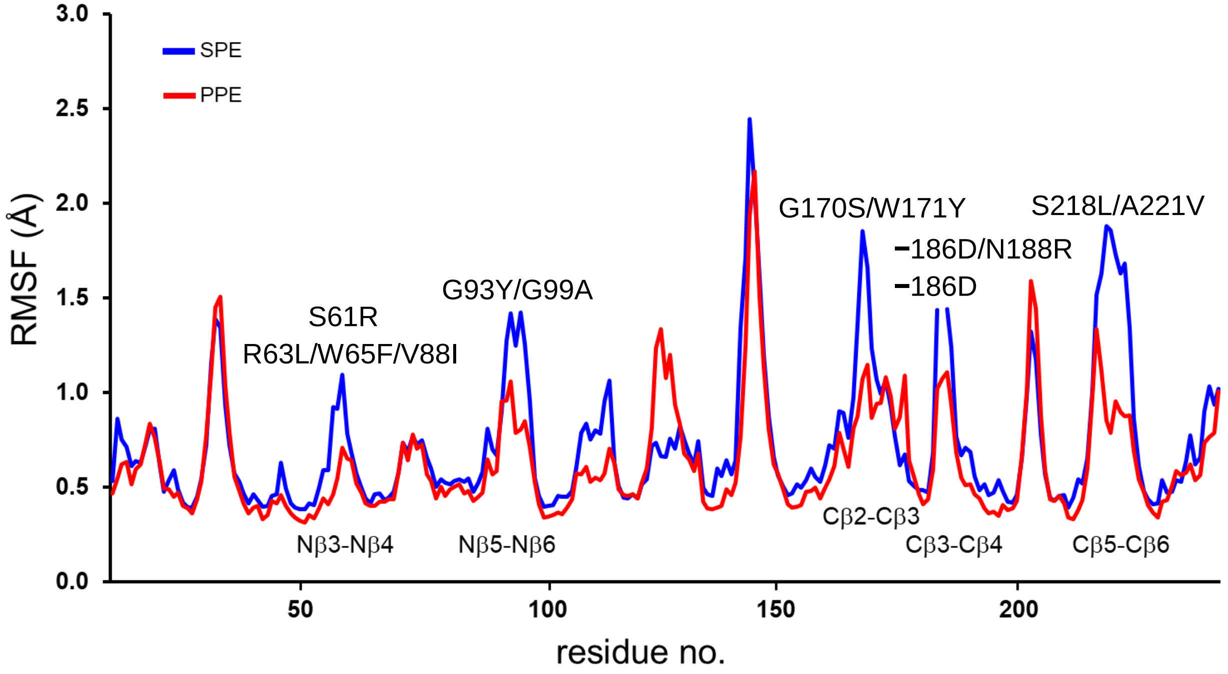

Supplement: Supplementary file 1 — Supplementary Information [file 41598_2019_55697_MOESM1_ESM.pdf]
